# Upfront Cranial Radiotherapy vs. EGFR Tyrosine Kinase Inhibitors Alone for the Treatment of Brain Metastases From Non-small-cell Lung Cancer: A Meta-Analysis of 1465 Patients

**DOI:** 10.3389/fonc.2018.00603

**Published:** 2018-12-12

**Authors:** Xiao-Jing Du, Su-Ming Pan, Shu-Zhen Lai, Xiao-Nan Xu, Mei-Ling Deng, Xiao-Hui Wang, Dun-Chen Yao, Shao-Xiong Wu

**Affiliations:** ^1^State Key Laboratory of Oncology in South China, Collaborative Innovation Center for Cancer Medicine, Department of Radiation Oncology, Sun Yat-sen University Cancer Center, Guangzhou, China; ^2^Department of Radiation Oncology, Yue Bei People's Hospital, Shaoguan, China

**Keywords:** non-small-cell lung cancer, whole brain radiation therapy, stereotactic radiosurgery, epidermal growth factor receptor, tyrosine kinase inhibitors

## Abstract

**Background:** Epidermal growth factor receptor tyrosine kinase inhibitors (EGFR-TKIs) is revolutionizing the management of brain metastases (BMs). This study was to explore the value of upfront cranial radiotherapy (RT) in EGFR-mutated non-small cell lung cancer (NSCLC) with BMs compared with EGFR-TKIs alone.

**Methods:** We searched all topic-related comparative articles in public databases (MEDLINE, EMBASE, Cochrane Library, and Web of Science) and conference proceedings. Outcomes of interest were intracranial objective response rate (ORR), overall survival (OS), and intracranial progression-free survival (PFS). Statistical analyses were calculated using Review Manager 5.3 software.

**Results:** Thirteen comparative studies that included a total of 1,456 patients were eligible. Upfront brain RT had significantly higher OS (HR = 0.78, 95% CI = 0.65–0.93, *P* = 0.005) than EGFR-TKI alone. Upfront RT plus TKI had superior OS (HR = 0.71, 95% CI = 0.58–0.86, *P* = 0.0005) and intracranial PFS (HR = 0.69, 95% CI = 0.49–0.99, *P* = 0.04). The pooled data favored upfront whole brain RT (WBRT) plus TKI in terms of intracranial PFS (HR = 0.64, 95% CI = 0.48–0.85, *P* = 0.002) and OS (HR = 0.75, 95% CI = 0.57–1, *P* = 0.05). Upfront stereotactic radiosurgery (SRS) was associated with better OS (HR = 0.37, 95% CI = 0.26–0.54, *P* < 0.00001). Similar results were observed when analysis was restricted to the use of erlotinib or geftinib.

**Conclusions:** The upfront use of brain RT seemed critical, especially for SRS. Upfront administration of upfront WBRT plus EGFR-TKI had better survival outcomes and seemed superior to EGFR-TKI alone.

## Key Points:

1. Treatment with first generation EGFR-TKIs (erlotinib or geftinib) alone was insufficient for BM management.
2. Significantly increased OS was observed in upfront RT groups compared with EGFR-TKI alone groups.

## Importance of the Study

Whether epidermal growth factor receptor tyrosine kinase inhibitors (EGFR-TKIs) alone is sufficient for patients with EGFR-mutated non-small-cell lung cancer (NSCLC) with BMs remains unclear. This meta-analysis shows significant improvement found after upfront brain radiotherapy compared with EGFR-TKI alone, especially for stereotactic radiosurgery. Upfront administration of upfront whole brain radiotherapy plus EGFR-TKI had better survival outcomes and seemed superior to EGFR-TKI alone.

## Introduction

Approximately 25 to 40% of all non-small-cell lung cancer (NSCLC) patients will develop brain metastases (BMs) during their disease course, and the risk is even higher in patients with epidermal growth factor receptor (EGFR) mutation (1). Brain radiotherapy (RT) that includes whole-brain radiotherapy (WBRT) and stereotactic radiosurgery (SRS) has been considered the state-of-the-art approach for BM management worldwide, but the survival benefit came at the cost of neurocognitive toxicity (2, 3).

Recently, strategies employed to treat metastatic NSCLC have evolved. EGFR tyrosine kinase inhibitors (EGFR-TKIs) have been integrated into the treatment algorithm of advanced metastatic EGFR-mutated NSCLC as first-line therapy, because of better response and survival rates compared with conventional chemotherapy (4). More importantly, EGFR-TKIs have shown promising intracranial activity in early-phase studies (5–8). Therefore, for EGFR-mutated NSCLC patients with newly developed BMs, it's of great interest to know whether the time of brain RT could be delayed after TKI administration alone to avoid the serious neurological side effects.

Several retrospective studies have reported survival benefits of upfront RT over TKI alone in treating EGFR-mutated NSCLC BMs (9–12). In 2015, a meta-analysis was conducted with 12 non-comparative observational studies including 363 patients. Although the strength of evidence was relatively low, the pooled data demonstrated that upfront brain RT (either WBRT or SRS) may improve survival outcomes but not intracranial disease response rates compared with TKI alone (13). Nevertheless, the only one randomized controlled trial (RCT) in this field so far, the BRAIN trial, has reported opposite result. In this trial, the majority of patients were with asymptomatic multiple BMs, and better survival outcomes was seen in patients receiving icotinib alone compared with upfront WBRT plus chemotherapy (14).

Thus, the optimal sequence of schedules for BM management remains controversial. In an attempt to address this question, we conducted the first meta-analysis of all topic-related comparative studies to explore the value of upfront RT compared with TKI alone in TKI treatment-naïve EGFR-mutated NSCLC patients with BM.

## Methods

This meta-analysis was conducted according to the Preferred Reporting Items for Systematic Reviews and Meta-Analysis (PRISMA) (15) and Meta-analysis Of Observational Studies in Epidemiology recommendations for study reporting (MOOSE) (16).

### Data Sources

A comprehensive literature search was performed using PubMed, EMBASE, Cochrane Library, and Web of Science from inception to 25 May 2018 without restrictions to language or region. We manually searched the annual meetings of ASCO, ASTRO, and the World Conference on Lung Cancer from 2008 onwards. The combinations of the following keywords were searched in [Mesh] and [Title/Abstract]: lung cancer/lung neoplasms/lung carcinoma/lung adenocarcinoma/NSCLC, irradiation/radiation therap^*^/radiotherap^*^/radiotherapeutics/radiation treatment/radiosurgery, brain metasta^*^/brain neoplasms/intracranialmetasta^*^/CNS metasta^*^leptomeningeal metasta^*^,erlotinib/tarceva/erbtinib/iressa/gefitinib/geftinat/icotinib/conmana/afatinib/dacomitinib/AZD9291/osimertinib/tagrisso/AZD3759/avitinib/rociletinib/CO-1686/olmutinib/nazartinib/EGF816/PF-06747775/tyrosine kinase inhibitor^*^/TKI^*^/EGFR-TKI^*^/targeted therapy.

After initially screening the title and abstract of the retrieved literatures, the full texts of relevant articles were independently assessed by two investigators for inclusion (X.J.D. and S.M.P.), and any disagreement was resolved by consensus. The related article function and manual searches of reference lists were also carried out to expand the included studies.

### Study Selection

The inclusion criteria for studies included in this meta-analysis were as follows: (1) pathologically confirmed NSCLC and newly diagnosed BMs with computed tomography (CT) or magnetic resonance imaging (MRI); (2) activated EGFR mutation status confirmed on genetic analysis; (3) EGFR-TKI treatment-naïve and no prior RT to the BMs; (4) compared upfront cranial RT vs. EGFR-TKI alone; (6) prospective cohorts, retrospective designs, or clinical trials were all included; (7) reported sufficient data on at least one of the outcomes: objective response rate (ORR) of intracranial disease (complete or partial response), overall survival (OS), or intracranial progression-free survival (PFS); and (8) response rate was determined using the Response Evaluation Criteria in Solid Tumors (RECIST) standards. Exclusion criteria were as follows: (1) case reports or case series with the number of study cases <5 in each arm; and (2) duplicate reports.

### Data Extraction

Two investigators (X.J.D. and S.M.P.) independently extracted and summarized the data from all included studies using a standardized data extraction form. For each study, the following items were extracted: first author, year of publication, type of study, country of origin study, intervention, sample size, ages, duration of follow up, and outcome measures. Any disagreement regarding data extraction was resolved by discussion and consensus among the investigators.

### Quality Assessment

The methodological quality of RCTs was assessed by the Cochrane risk of bias tool based on the following criteria: (1) random sequence generation; (2) allocation concealment; (3) blinding of participants and personnel; (4) blinding of outcome assessment; (5) incomplete outcome data; (6) selective reporting; and (7) other bias (17). Each trial for bias based on the above-listed criteria was marked as low, high, or unclear risk. The quality was defined as follows: A rating, meeting all criteria of low risk; B rating, meeting one or more criteria of unclear risk without high risk; C rating, meeting one or more criteria of high risk. The methodological quality of retrospective studies was appraised using the Newcastle-Ottawa scale (18), which comprised the three following items: patient selection, comparability of the study groups, and assessment of outcomes. The quality of each retrospective study was scored on a scale ranging from 0 to 9 by two independent investigators. Studies with scores ≥6 were regarded as high-quality.

### Statistical Methods

The odds ratio (OR) with 95% confidence interval (95% CI) was calculated with regards to ORR. Hazard ratios (HRs) and 95% confidence intervals (CIs) were used as summary statistics for time-to-event data, which were directly extracted from the research article or calculated using previously published methods, as proposed by Tierney et al (19).

The meta-analysis and forest plots were produced by Review Manager 5.3 (Cochrane Collaboration, Oxford, UK). Statistical heterogeneity between studies was appraised using the Chi-square (χ2) and I-square (I^2^) test. No heterogeneity existed when P > 0.1 and I^2^ < 50%. A fixed-effect model was applied to pool the study results. Significant heterogeneity was found if P < 0.1 and I^2^ > 50%, and a random-effects statistical model was used (20).

Subgroup analyses were performed to explore the optimal modality of treatment. Sensitivity analysis was conducted to assess the stability of the study results. The results were analyzed after individual exclusion, i.e., one study each time, from the meta-analysis. Funnel plots were performed to detect potential publication bias.

## Results

### Included Studies

**Figure 1:**
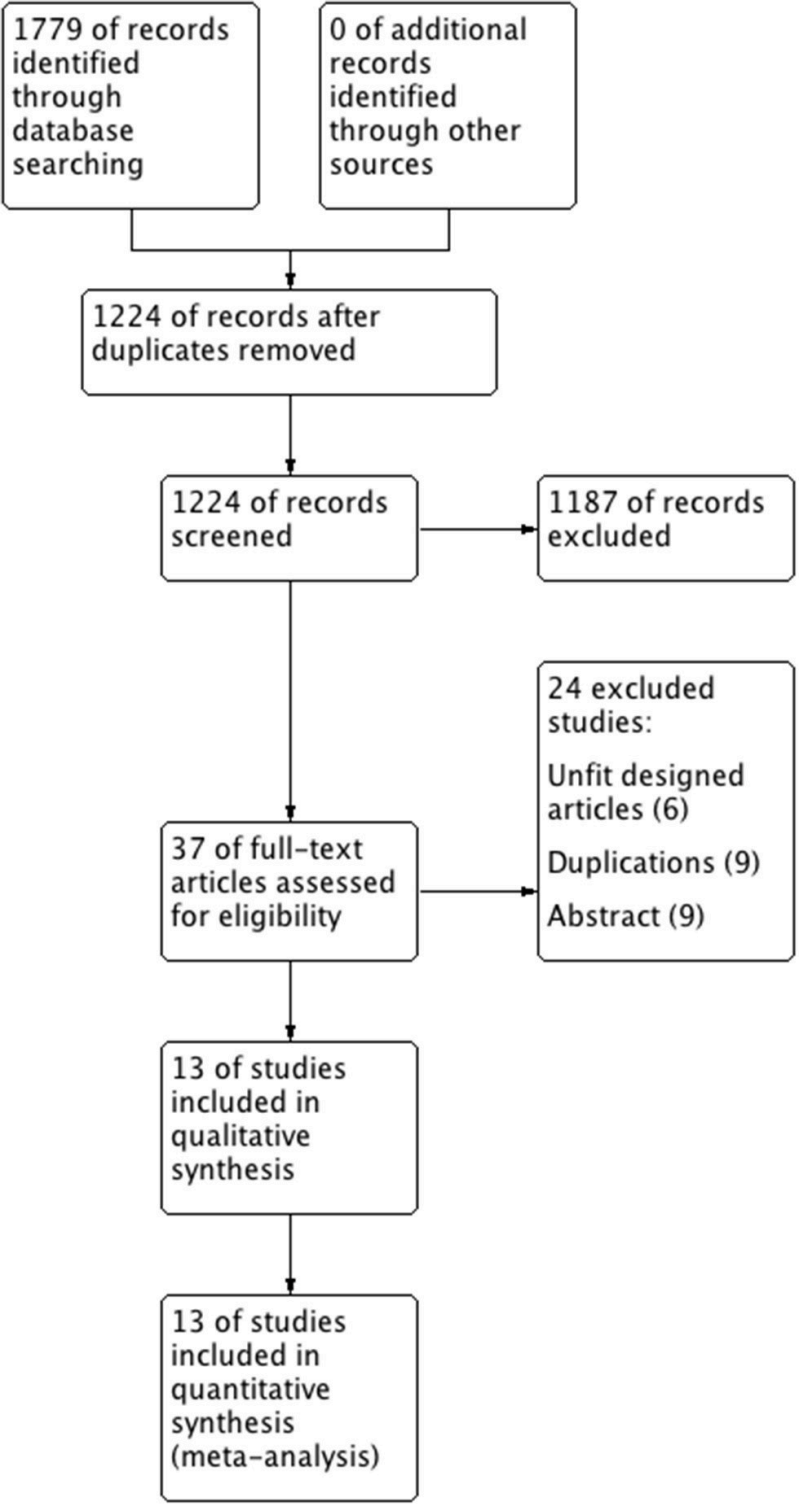
Flowchart showing the selection of the trials.

The literature selection process is presented in the PRISMA flow chart (Figure 1) according to the PRISMA guidelines. After comprehensive discussion and analysis, 13 studies were selected and included in the final meta-analysis. A total of 1,456 patients with BMs originated from EGFR-mutated NSCLC in the included studies. Table 1 presents the baseline characteristics of the eligible studies. Twelve of the 13 eligible articles were retrospective studies (9–12, 21–28); the remaining one was a RCT (14). Eleven of them used upfront RT plus TKI in the treatment group (9–12, 21, 23–28), whereas the other two defined their treatment group just as upfront RT (14, 22). All studies were published in English; 10 studies were performed in Asia (10, 12, 14, 21, 23–28). One study did not report the median follow-up (25), whereas all the other 12 studies reported the follow-up, which lasted more than 16 months.

**Table 1:** Characteristics of included studies.

**Studies**	**Country**	**Study design**	**Time range**	**Samples (T/C)^*†*^**	**Age (T/C)^‡^, years**	**Treatment group**	**Control group**	**Median follow-up, mo**.	**Outcomes**	**Study quality**
Zeng et al. (21)	Mainland, China	R	2005–2009	12 (7/5)	NR	WBRT+TKI	Gefitinib	23	RR, DCR	6
Gerber et al. (22)	America	R	2006–2012	110 (32/15/63)	58 ± 11/61 ± 11/62 ± 13	WBRT/SRS	erlotinib	20	OS, iPFS	7
Byeon et al. (23)	South Korea	R	2005–2013	121 (59/62)	60/60	WBRT/SRS+TKI	Erlotinib/gefitinib	18.4	DCR, OS, iPFS	7
Chen et al. (24)	Mainland, China	R	2008–2014	132 (53/79)	52/52	WBRT+TKI	Erlotinib/gefitinib	36.2	RR, OS, iPFS	7
Jiang et al. (25)	Mainland, China	R	2012–2015	121(30/91)	NR	WBRT+TKI	Erlotinib/gefitinib/icotinib	NR	OS, iPFS	5
Magnuson et al. (9)	America	R	2008–2014	50 (33/17)	59/60	WBRT/SRS+TKI	erlotinib	20.6	RR, OS, iPFS	7
Fan et al. (26)	Mainland, China	R	2011–2014	97 (56/41)	56/59	WBRT/SRS+TKI	icotinib	28.5	RR, OS, iPFS	7
Liu et al. (10)	Mainland, China	R	2008–2015	71 (NR)	NR	WBRT/SRS+TKI	Erlotinib/gefitinib/icotinib	30	OS	6
Magnuson (11)	America	R	2008–2014	351 (120/100/131)	58/63/60	WBRT/SRS+TKI	TKI	22	OS, iPFS	7
Yang et al. (14)	Mainland, China	RCT	2012–2015	158 (73/85)	58/57	WBRT	icotinib	16.5	RR, DCR, OS, iPFS	C
Zhu et al. (12)	Mainland, China	R	2011–2015	133 (67/66)	56	WBRT/SRS+TKI	Erlotinib/gefitinib	18	OS, iPFS	7
Li et al. (27)	Taiwan, China	R	2014–2016	28 (17/11)	57.3 ± 9.4/62.0 ± 7.3	WBRT+TKI	afatinib	17.4	RR, DCR, OS, iPFS	7
Sung et al. (28)	South Korea	R	2008–2016	81 (40/41)	64	WBRT/SRS+TKI	Erlotinib/gefitinib	20	RR, DCR, OS, iPFS	7

*R, retrospective; RCT, randomized control trial; mo., months; NR, not reported; T, treatment group (upfront radiotherapy); C, control group (TKI alone); WBRT, whole brain radiotherapy; SRS, stereotactic radiosurgery; TKI, tyrosine kinase inhibitors; ORR, objective response rate; DCR, disease control rate; OS, overall survival; iPFS, intracranial progression-free survival*.

† *The sample size is presented as “WBRT/SRS/TKI alone” or “WBRT or SRS/TKI alone,” according to the original study design of the included studies*.

‡ *The age is presented as “median age” or “mean age ±SD” for each group or the entire cohort*.

### Methodological Quality

The general quality of the 12 retrospective studies was fair. Eleven of the 12 studies had scores of ≥6 (Table S1). As for the RCT, the study complied with the intention-to-treat principle, avoided selective outcome reporting, and assessed each outcome adequately. Random method was used. The implementation of allocation concealment was not described. Furthermore, clinicians and patients were not blinded to the treatment allocation. Therefore, this study received C quality score (Table S2).

### Intracranial Objective Response Rate

Seven of the eligible studies reported the intracranial objective response rate (ORR) of treatment using upfront RT and TKI alone (9, 14, 21, 24, 26–28). ORR ranged from 37.0 to 88.2% in the upfront RT groups and from 39 to 81.8% in the TKI alone groups.

**Figure 2:**
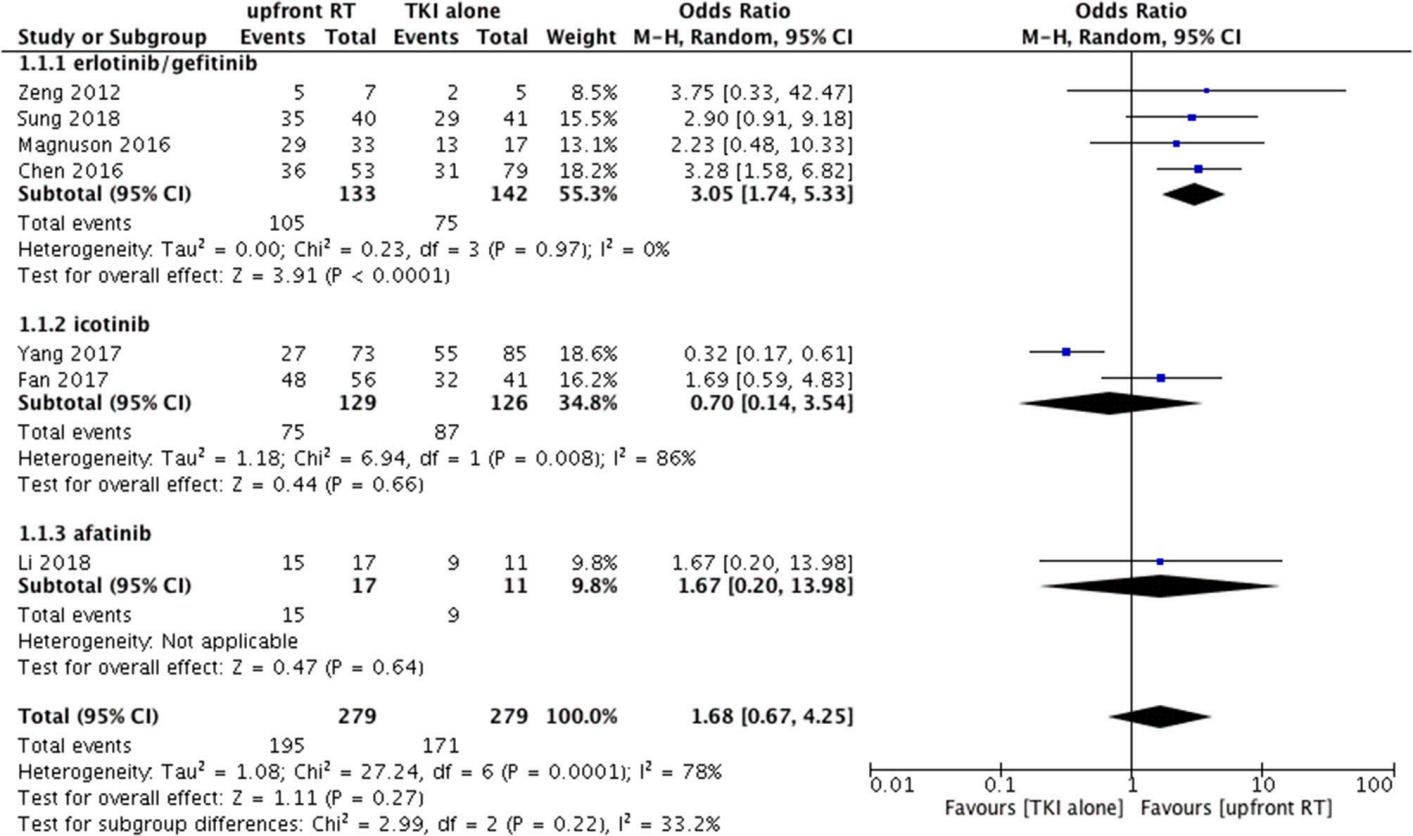
Forest plot and meta-analysis of intracranial objective response rate (ORR) in subgroup analysis.

Heterogeneity was observed among the eligible studies in ORR (*P* = 0.0001, I^2^ = 78%). As a result, the random-effects model and the subgroup analysis stratified by the type of TKI were used for the meta-analysis (Figure 2). The pooled results indicated that upfront RT resulted in superior intracranial ORR when compared with erlotinib/geftinib alone groups (OR = 3.05, 95% CI: 1.74–5.33, *P* < 0.0001), with no heterogeneity (*P* = 0.97, I^2^ = 0%) (9, 21, 24, 28). For the icotinib alone groups, studies included one RCT and one retrospective cohort study (14, 26); apparent heterogeneity in the ORR meta-analysis was observed (*P* = 0.008, I^2^ = 86%). The pooled results showed that no significant difference was found between upfront RT groups and icotinib alone groups (OR = 0.7, 95% CI: 0.14–3.54, *P* = 0.66) (Figure 2).

### Overall Survival

#### RT vs. TKI Alone

A total of 12 trials reported OS (9–12, 14, 22–28). Only one of the two studies were included in the meta-analysis because of data duplication published by the same author with a coincident study period (9, 11). Among the 11 eligible studies, 6 studies used upfront WBRT as the treatment group (11, 14, 22, 24, 25, 27). In the other 5 studies, the treatment group was defined as upfront RT (either WBRT or SRS) (10, 12, 23, 26, 28). A fixed-effect model was utilized for the meta-analysis, because heterogeneity did not exist (*P* = 0.22; I^2^ = 24%). Notably, pooled data revealed a significant difference in terms of OS between the groups of patients who were treated with upfront RT and those who received TKI alone (HR = 0.78, 95% CI: 0.65–0.93, *P* = 0.005).

**Figure 3:**
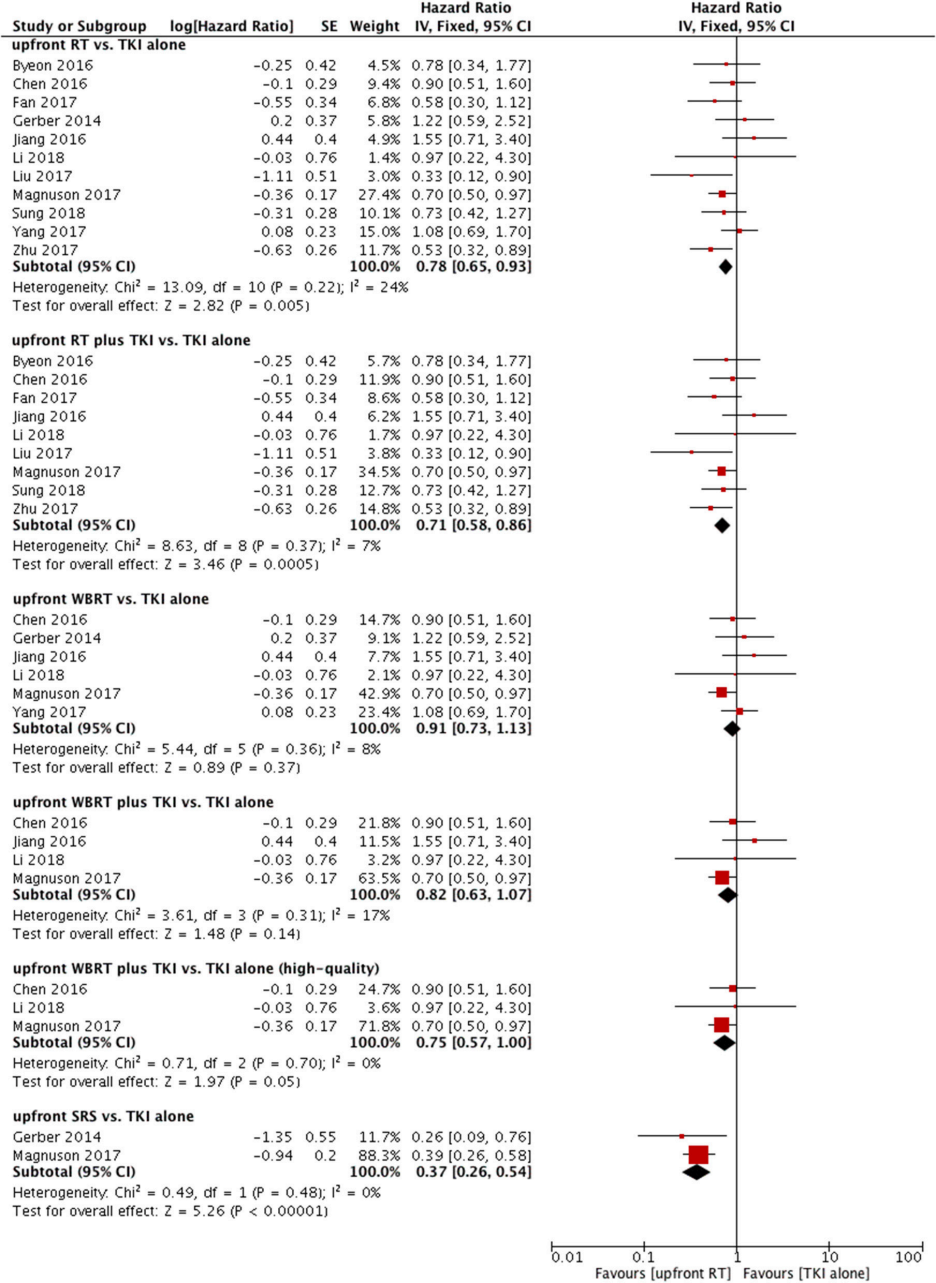
Forest plot and meta-analysis of overall survival (OS). RT, radiotherapy; WBRT, whole brain radiotherapy; SRS, stereotactic radiosurgery; TKI, tyrosine kinase inhibitor.

Similarly, significant difference in OS was also observed when comparing upfront RT plus TKI with TKI alone (HR = 0.71, 95% CI: 0.58–0.86, *P* = 0.0005) (10–12, 23–28) without heterogeneity (*P* = 0.37; I^2^ = 7%) (Figure 3).

#### WBRT vs. TKI Alone

Six studies compared OS between upfront WBRT and TKI alone (11, 14, 22, 24, 25, 27); these studies did not show heterogeneity (*P* = 0.36, I^2^ = 8%). Analysis using a fixed-effect model suggested that no significant difference was found (HR = 0.91, 95% CI: 0.73–1.13, *P* = 0.37).

Similar results were observed in the comparison between upfront WBRT plus TKI groups and TKI alone groups (HR = 0.82, 95% CI: 0.63–1.07, *P* = 0.14) (Figure 3) (11, 24, 25, 27). However, in the sensitivity analysis, the results indicated a superior OS in the upfront WBRT plus TKI group with marginally significance (HR = 0.75, 95% CI: 0.57–1, *P* = 0.05) without heterogeneity (*P* = 0.70, I^2^ = 0%) when one study with relatively low-quality (25) was omitted from the analysis. This particular study was lack of detailed information between different treatment groups as well as follow-up data, raising concerns of potential bias.

#### SRS vs. TKI Alone

Two studies reported OS comparing upfront SRS with TKI alone (11, 22); these studies did not show heterogeneity (*P* = 0.48, I^2^ = 0%). A fixed-effect model was applied. The outcome suggested that compared with TKI alone, upfront SRS possessed superior OS for patients (HR = 0.37, 95% CI: 0.26–0.54, *P* < 0.00001) (Figure 3).

### Erlotinib/geftinib Subgroup

**Figure 4:**
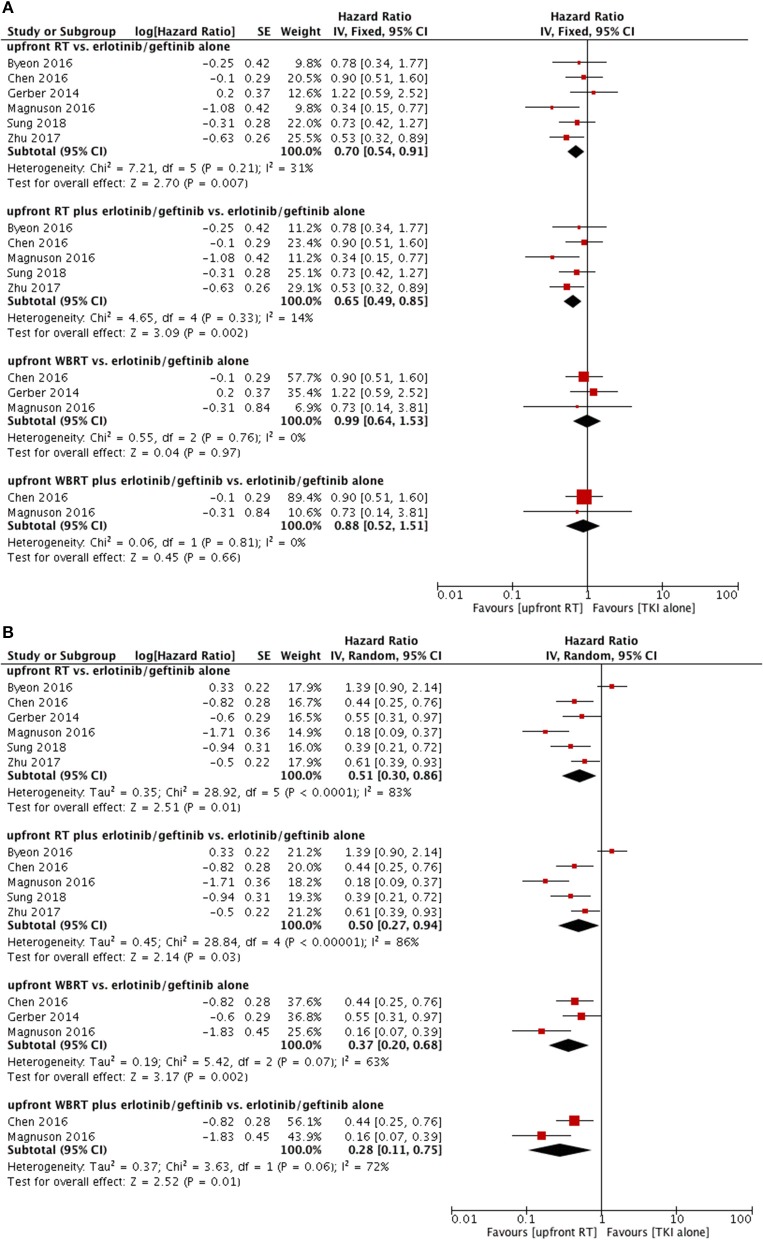
Forest plot and meta-analysis for upfront radiotherapy vs. erlotinib/geftinib alone. **(A)** Overall survival. **(B)** Intracranial progression-free survival. RT, radiotherapy; WBRT, whole brain radiotherapy.

As for the erlotinib/geftinib groups, pooled data indicated superior OS in the upfront RT groups (HR = 0.70, 95% CI: 0.54–0.91, *P* = 0.007) (9, 12, 22–24, 28) and upfront RT plus TKI groups (HR = 0.65, 95% CI: 0.49–0.85, *P* = 0.002) (9, 12, 23, 24, 28) but not in the upfront WBRT groups (9, 22, 24) or upfront WBRT plus TKI groups (9, 24); no heterogeneity was found (Figure 4A).

### Intracranial Progression-Free Survival

#### RT vs. TKI Alone

A total of 10 studies were eligible in the meta-analysis for intracranial PFS (11, 12, 14, 22–28). A random-effects model was applied based on the heterogeneity values (*P* < 0.00001, I^2^ = 78%). There was no significant intracranial PFS difference between upfront RT and TKI groups (HR = 0.75, 95% CI: 0.53–1.06, *P* = 0.11) (Figure S1).

**Figure 5:**
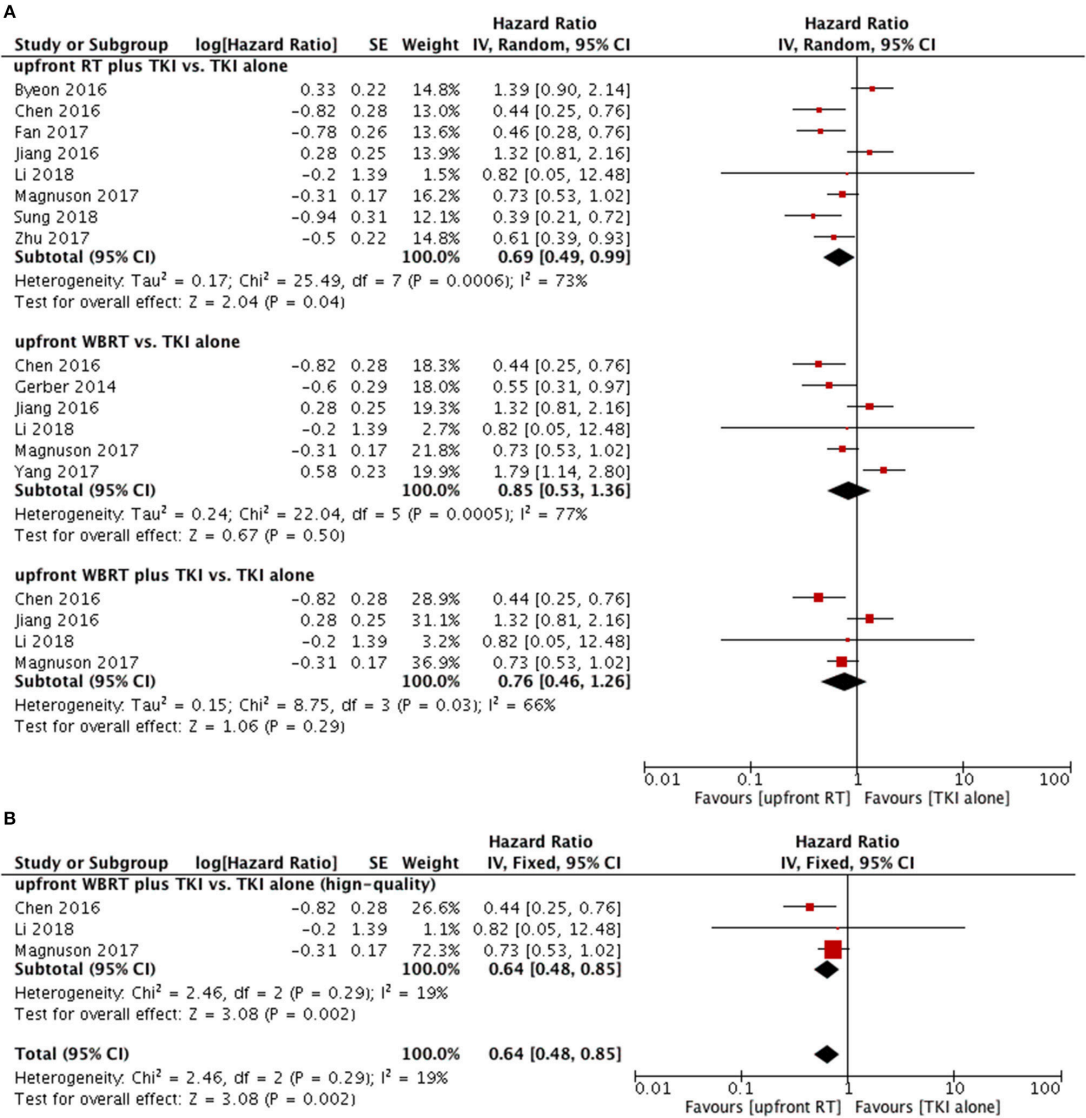
Forest plot and meta-analysis of intacranial progression-free survival (PFS). **(A)** Pooled data based on random-effects model. **(B)** Pooled data based on fix-effect model. RT, radiotherapy; WBRT, whole brain radiotherapy; TKI, tyrosine kinase inhibitor.

Intracranial PFS in upfront RT plus TKI groups was superior to that of TKI alone groups (HR = 0.69, 95% CI: 0.49–0.99, *P* = 0.04) (11, 12, 23–28) despite of existence of heterogeneity (*P* = 0.0006, I^2^ = 73%) (Figure 5A). However, the sensitivity analysis failed to demonstrate a robust result regarding this comparison.

#### WBRT vs. TKI Alone

Subgroup analyses did not show differences in intracranial PFS between upfront WBRT groups and TKI alone groups (HR = 0.85, 95% CI: 0.53–1.36, *P* = 0.5) (Figure 4A) (11, 14, 22, 24, 25, 27).

As for upfront WBRT plus TKI groups vs. TKI alone groups (11, 24, 25, 27), the pooled data also did not show a significant difference in intracranial PFS (HR = 0.76, 95% CI: 0.46–1.26, *P* = 0.29). In the sensitivity analysis, the results changed to favor the upfront WBRT plus TKI group (HR = 0.64, 95% CI: 0.48–0.85, *P* = 0.002) without heterogeneity (*P* = 0.29, I^2^ = 19%) when the study with relatively low-quality (25) was omitted from the meta-analysis and a fix-effect model was applied (Figure 5B).

#### Asymptomatic BMs Subgroup

**Figure 6:**
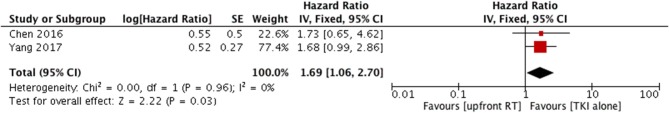
Forest plot and meta-analysis of intacranial progression-free survival (PFS) f in patients with asymptomatic brain metastases. RT, radiotherapy; TKI, tyrosine kinase inhibitor.

Two studies compared intracranial PFS between upfront WBRT and TKI alone (14, 24); they did not show heterogeneity (*P* = 0.96, I2 = 0%). Analysis using a fixed-effect model suggested that a superior PFS in the TKI alone group (HR = 1.69, 95% CI: 1.06–2.7, *P* = 0.03) (Figure 6).

#### Erlotinib/geftinib Subgroup

For the erlotinib/gefitinib alone groups, the outcomes revealed significant superior intracranial PFS in upfront RT groups (HR = 0.51, 95% CI: 0.30–0.86, *P* = 0.01) (9, 12, 22–24, 28), upfront RT plus TKI groups (HR = 0.50, 95% CI: 0.27–0.94, *P* = 0.03) (9, 12, 23, 24, 28), upfront WBRT groups (HR = 0.37, 95% CI: 0.20–0.68, *P* = 0.002) (9, 22, 24), and upfront WBRT plus TKI groups (HR = 0.28, 95% CI: 0.11–0.75, *P* = 0.01) (9, 24) despite the existence of heterogeneity (Figure 4B). Sensitivity analysis showed that the omission of any single study did not significantly affect these results.

### Publication Bias

**Figure 7:**
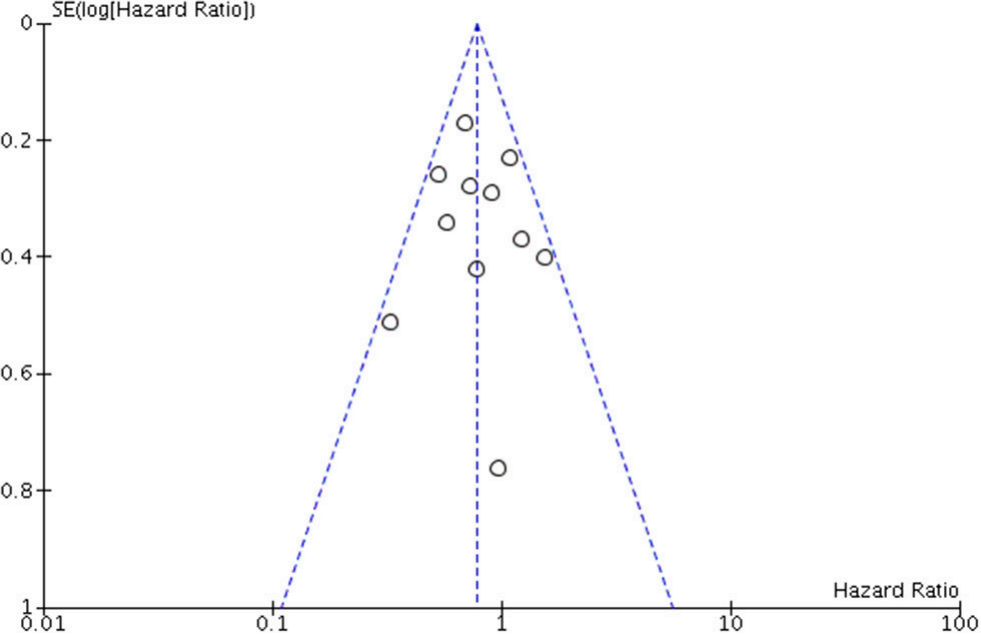
Funnel plot for assessing publication bias of overall survival (OS) in overall meta-analysis.

A funnel plot was created for OS. Scatters of all the studies were mainly concentrated on both sides of the straight line and close to the tip of the funnel with a symmetric distribution, thereby suggesting that no obvious publication bias existed (Figure 7).

## Discussion

With the recent progress in novel EGFR-TKIs, a fervent debate on the omission or delay of brain RT for EGFR-mutated NSCLC BMs by using TKIs alone has been rekindled (29–31). To our knowledge, this is the largest meta-analysis that evaluated the efficacy of upfront RT vs. EGFR-TKI alone in management of TKI-naïve EGFR-mutated NSCLC BMs by using all comparative studies.

Compared with TKI alone, upfront RT significantly improved OS but not intracranial ORR or PFS. Since WBRT is widely used for multiple BMs and SRS is always for oligo BMs providing more focal and aggressive radiation as well as normal tissue sparing, the difference of intracranial tumor burden between this two RT groups is not surprising (Table S3). When separated by type of RT, the patients treated with upfront SRS tended to have better OS. On the contrary, no significant difference in outcomes was observed regarding upfront WBRT and TKI alone. Most eligible studies were retrospective. Thus, the results were partially attributed to the imbalance of inclusion criteria between the upfront WBRT groups and TKI alone groups. For example, patients with symptomatic and multiple BMs were more often treated with upfront WBRT in real-world practice, whereas patients receiving TKI alone more likely presented asymptomatic intracranial disease and had smaller BMs (Table S4). Notably, a large retrospective study including multicenter data demonstrated that the WBRT group had longer OS compared with the TKI alone group (52 vs. 32 months) when analysis was restricted to patients all with favorable disease-specific Graded Prognostic Assessment (dsGPA 2–4) (11). However, Chen et al. reported better intracranial PFS obtained by upfront WBRT in patients with symptomatic BMs in the subgroup analysis of their study (24). Further analysis based on intracranial tumor burden or disease severity is unavailable by now. Clinical trials are needed to further illustrate this issue.

Administration of brain RT together with EGFR-TKI is worth exploring. Several mechanisms of synergistic action between EGFR inhibition and brain RT has been proposed, including disturbing cell cycle kinetics, apoptosis induction, and inhibition of radiation-induced activation of EGFR (2, 32). Furthermore, WBRT can open the blood–brain barrier by damaging the endothelial cells, and significantly increase TKI's exposure in the cerebrospinal fluid (32, 33). Theoretically, the blood-brain barrier might be repaired ~3 weeks after brain RT; the permeability changes after RT depend on the timing of the TKI administration (32–34). Thus, concurrent or early-started adjuvant TKI therapy combined with brain RT might be an ideal treatment regimen for EGFR-mutated patients that did not receive prior TKI treatment. A recent meta-analysis also demonstrated that RT plus EGFR-TKIs was more effective in improving ORR and disease control rate than RT alone (35). In accordance with these findings, we observed an improvement in OS and potentially in intracranial PFS in the combined therapy groups.

Erlotinib and geftinib are the two most popular first generation EGFR-TKIs. Thus, a meta-analysis restricted to studies that included patients who received erlotinib/geftinib was also conducted. We observed better intracranial ORR, intracranial PFS, and OS in patients treated with upfront RT compared with erlotinib/geftinib alone. A potential improvement in intracranial PFS was also demonstrated in the upfront WBRT groups. Actually, pharmacological studies showed that the penetrative ability to cross the blood–brain barrier seemed limited to these two drugs, thereby implying that erlotinib/geftinib alone may not be very effective in the management of BMs (31).

In the BRAIN trial (14), the intracranial ORR in the icotinib alone group was 65%, which was comparable with the data of erlotinib/geftinib alone (ORR 39–76%) (9, 21, 24, 28) and slightly lower than the data of the same TKI in retrospective study (ORR 78%) (26). Two facts could partially explain the significant superior PFS obtained in the icotinib group. First, the targeted population in BRAIN were patients with multiple (≥3) BMs, and more than 80% of them were asymptomatic, which might decrease the urgency of brain RT and kindly help TKIs to give full play to their role in the treatment. Our subgroup meta-analysis in asymptomatic patients has partially confirmed this suspicion. Secondly, contrary to the first-line treatment recommendation for metastatic NSCLC, no concurrent or adjuvant TKI therapy was applied to the upfront WBRT group according to the study design, which might weaken the efficacy of RT group. In fact, 11 of 13 included studies had set the interventional group as brain RT combined with TKI. It was remarkable that the intracranial ORR in the upfront WBRT group was only 37% in Brain trial, which was significantly lower than the data reported from other studies (ORR 68–88.2%) (21, 24, 27).

New generation EGFR-TKIs have been designed for clinical application, such as afatinib, AZD9291, and AZD3759. These TKIs showed impressive intracranial penetration into the cerebrospinal fluid according to several preclinical or early-phase studies (7, 8, 36); such activity was better than that of first generation EGFR-TKIs. Nevertheless, the results from ongoing trials are worth waiting for (ClinicalTrials.gov identifier: NCT02714010, NCT02768337, NCT02972333, NCT02736513).

The main limitation of the present meta-analysis is the inferior level of evidence with only one RCT of level C quality. The remaining 12 studies were retrospective comparative articles with relatively high risk of selection bias in the treatment arm and in the control arm. Subgroup meta-analysis stratified by different treatment regimens analyzed only a limited number of eligible studies and a relatively small number of patients. In addition, the random effects model used in a part of the intracranial PFS analysis may increase the effect of a small sample with unsatisfactory quality.

## Conclusions

This meta-analysis reports that upfront RT significantly increased OS compared with EGFR-TKI alone, especially when upfront SRS is applied to the treatment of limited BMs in EGFR-mutated NSCLC. In addition, it is reasonable to combine EGFR-TKIs and WBRT in the treatment of multiple BMs. Treatment with first generation EGFR-TKIs (erlotinib or geftinib) alone seems insufficient for BM management in this group of patients. RCTs are needed to further explore this issue.

## Author Contributions

S-XW and X-JD conception and design. X-JD and S-XW protocol development. X-JD, S-MP, S-ZL, and X-NX acquisition of data (study selection, data extraction, etc.). X-JD, S-MP, S-ZL, X-NX, X-HW, D-CY, and M-LD analysis and interpretation of data. X-JD and S-XW writing, review, and/or revision of the manuscript. S-XW study supervision.

### Conflict of Interest Statement

The authors declare that the research was conducted in the absence of any commercial or financial relationships that could be construed as a potential conflict of interest.

## Supplementary Material

Table S1 Quality assessment of 12 retrospective studies using the Newcastle-Ottawa scale.

Table S2 Quality assessment of one randomized controlled trial.

Table S3 Comparing the tumor burden between WBRT group and SRS group.

Table S4 Detailed characteristics for each included study.

Figure S1 Forest plot and meta-analysis of intacranial progression-free survival (PFS) comparing upfront RT with TKI alone. RT, radiotherapy; TKI, tyrosine kinase inhibitor.

## References

[1] Li LN Luo SM Lin H Yang HT Chen HJ Liao ZY. Correlation between EGFR mutation status and the incidence of brain metastases in patients with non-small cell lung cancer. J Thorac Dis. (2017) 9:2510–20. 10.21037/jtd.2017.07.57 28932557 PMC5594201

[2] Khalifa J Amini A Popat S Gaspar LE Faivre-Finn C. Brain metastases from NSCLC: radiation therapy in the era of targeted therapies. J Thorac Oncol. (2016) 11:1627–43. 10.1016/j.jtho.2016.06.002 27343440

[3] Soffietti R Abacioglu U Baumert B Combs SE Kinhult S Kros JM. Diagnosis and treatment of brain metastases from solid tumors: guidelines from the european association of neuro-oncology (EANO). Neuro Oncol. (2017) 19:162–74. 10.1093/neuonc/now241 28391295 PMC5620494

[4] Reck M Popat S Reinmuth N De Ruysscher D Kerr KM Peters S. Metastatic non-small-cell lung cancer (NSCLC): ESMO clinical practice guidelines for diagnosis, treatment and follow-up. Ann Oncol. (2014) 25 (Suppl. 3):27–39. 10.1093/annonc/mdu199 25115305

[5] Kim JE Lee DH Choi Y Yoon DH Kim SW Suh C. Epidermal growth factor receptor tyrosine kinase inhibitors as a first-line therapy for never-smokers with adenocarcinoma of the lung having asymptomatic synchronous brain metastasis. Lung Cancer (2009) 65:351–4. 10.1016/j.lungcan.2008.12.011 19157632

[6] Iuchi T Shingyoji M Sakaida T Hatano K Nagano O Itakura M. Phase II trial of gefitinib alone without radiation therapy for Japanese patients with brain metastases from EGFR-mutant lung adenocarcinoma. Lung Cancer (2013) 82:282–7. 10.1016/j.lungcan.2013.08.016 24021541

[7] Hoffknecht P Tufman A Wehler T Pelzer T Wiewrodt R Schütz M Efficacy of the irreversible ErbB family blocker afatinib in epidermal growth factor receptor (EGFR) tyrosine kinase inhibitor (TKI)-pretreated non-small-cell lung cancer patients with brain metastases or leptomeningeal disease. J Thorac Oncol. (2015) 10:156–63. 10.1097/JTO.0000000000000380 25247337 PMC4276567

[8] Ahn MJ Kim DW Cho BC Kim SW Lee JS Ahn JS. Activity and safety of AZD3759 in EGFR-mutant non-small-cell lung cancer with CNS metastases (BLOOM): a phase 1, open-label, dose-escalation and dose-expansion study. Lancet Respir Med. (2017) 5:891–902. 10.1016/S2213-2600(17)30378-8 29056570

[9] Magnuson W Yeung J Guillod P Gettinger S Yu J Chiang V. Impact of deferring radiation therapy in patients with epidermal growth factor receptor-mutant non-small cell lung cancer who develop brain metastases. Int J Radiat Oncol Biol Phys. (2016) 95:673–9. 10.1016/j.ijrobp.2016.01.037 27034176

[10] Liu Y Deng L Zhou X Gong Y Xu Y Zhou L. Concurrent brain radiotherapy and EGFR-TKI may improve intracranial metastases control in non-small cell lung cancer and have survival benefit in patients with low DS-GPA score. Oncotarget (2017) 8:111309–17. 10.18632/oncotarget.22785 29340055 PMC5762323

[11] Magnuson W Lester-Coll N Wu A Yang T Lockney N Gerber N. Management of brain metastases in tyrosine kinase inhibitor-Naive epidermal growth factor receptor-mutant non-small-cell lung cancer: a retrospective multi-institutional analysis. J Clin Oncol. (2017) 35:1070–1077. 10.1200/JCO.2016.69.7144 28113019

[12] Zhu Q Sun Y Cui Y Ye K Yang C Yang D. Clinical outcome of tyrosine kinase inhibitors alone or combined with radiotherapy for brain metastases from epidermal growth factor receptor (EGFR) mutant non small cell lung cancer (NSCLC). Oncotarget (2017) 8:13304–11. 10.18632/oncotarget.14515 28076323 PMC5355097

[13] Soon YY Leong CN Koh WY Tham IW. EGFR tyrosine kinase inhibitors versus cranial radiation therapy for EGFR mutant non-small cell lung cancer with brain metastases: a systematic review and meta-analysis. Radiother Oncol. (2015) 114:167–72. 10.1016/j.radonc.2014.12.011 25583566

[14] Yang JJ Zhou CC Huang YS Feng JF Lu S Song Y. Icotinib versus whole-brain irradiation in patients with EGFR-mutant non-small-cell lung cancer and multiple brain metastases (BRAIN): a multicentre, phase 3, open-label, parallel, randomised controlled trial. Lancet Respir Med. (2017) 5:707–16. 10.1016/S2213-2600(17)30262-X 28734822

[15] Moher D Shamseer L Clarke M Ghersi D Liberati A Petticrew M. Preferred reporting items for systematic review and meta-analysis protocols (PRISMA-P) 2015 statement. Syst Rev. (2015) 4:1. 10.1186/2046-4053-4-1 25554246 PMC4320440

[16] Stroup DF Berlin JA Morton SC Olkin I Williamson GD Rennie D. Meta-analysis of observational studies in epidemiology: a proposal for reporting. Meta-analysis of observational studies in epidemiology (MOOSE) group JAMA (2000) 283:2008–12. 10789670 10.1001/jama.283.15.2008

[17] Higgins J Green S Cochrane Handbook for Systematic Reviews of Interventions Version 5.1.0. New York, NY: Cochrane Collaboration, John Wiley and Sons (2011). Available online at: http://handbook.cochrane.org/ (Accessed May, 2018).

[18] Wells GA Shea BJ O'Connell D Peterson J Welch V Losos M The Newcastle-Ottawa Scale (NOS) for Assessing the Quality of Non-randomised Studies in Meta-Analyses (2000). Available Online at: www.ohri.ca/programs/clinical_epidemiology/nos_manual.doc

[19] Tierney JF Stewart LA Ghersi D Burdett S Sydes MR. Practical methods for incorporating summary time-to-event data into meta-analysis. Trials (2007) 8:16. 10.1186/1745-6215-8-16 17555582 PMC1920534

[20] Higgins JP Thompson SG. Quantifying heterogeneity in a meta-analysis. Stat Med. (2002) 21:1539–58. 10.1002/sim.1186 12111919

[21] Zeng YD Zhang L Liao H Liang Y Xu F Liu JL. Gefitinib alone or with concomitant whole brain radiotherapy for patients with brain metastasis from non-small-cell lung cancer: a retrospective study. Asian Pac J Cancer Prev. (2012) 13:909–14. 10.7314/APJCP.2012.13.3.909 22631670

[22] Gerber NK Yamada Y Rimner A Shi W Riely GJ Beal K. Erlotinib versus radiation therapy for brain metastases in patients with EGFR-mutant lung adenocarcinoma. Int J Radiat Oncol Biol Phys. (2014) 89:322–9. 10.1016/j.ijrobp.2014.02.022 24679729 PMC5691362

[23] Byeon S Ham JS Sun JM Lee SH Ahn JS Park K. Analysis of the benefit of sequential cranial radiotherapy in patients with EGFR mutant non-small cell lung cancer and brain metastasis. Med Oncol. (2016) 33:97. 10.1007/s12032-016-0811-3 27447711 PMC4958121

[24] Chen Y Yang J Li X Hao D Wu X Yang Y. First-line epidermal growth factor receptor (EGFR)–tyrosine kinase inhibitor alone or with whole-brain radiotherapy for brain metastases in patients with EGFR-mutated lung adenocarcinoma. Cancer Sci. (2016) 107:1800–5. 10.1111/cas.13079 27627582 PMC5198957

[25] Jiang T Su C Li X Zhao C Zhou F Ren S. EGFR TKIs plus WBRT demonstrated no survival benefit other than that of TKIs alone in patients with NSCLC and EGFR mutation and brain metastases. J Thorac Oncol. (2016) 11:1718–28. 10.1016/j.jtho.2016.05.013 27237825

[26] Fan Y Xu Y Gong L Fang L Lu H Qin J. Effects of icotinib with and without radiation therapy on patients with EGFR mutant non-small cell lung cancer and brain metastases. Sci Rep. (2017) 7:45193. 10.1038/srep45193 28332624 PMC5362911

[27] Li SH Liu CY Hsu PC Fang YF Wang CC Kao KC. Response to afatinib in treatment-naïve patients with advanced mutant epidermal growth factor receptor lung adenocarcinoma with brain metastases. Exp Rev Anticancer Ther. (2018) 18:81–9. 10.1080/14737140.2018.1409623 29172778

[28] Sung S Lee SW Kwak YK Kang JH Hong SH Kim YS. Intracranial control and survival outcome of tyrosine kinase inhibitor (TKI) alone versus TKI plus radiotherapy for brain metastasis of epidermal growth factor receptor-mutant non-small cell lung cancer. J Neurooncol. (2018) 139:205–13 10.1007/s11060-018-2861-1 29644484

[29] Martínez P Mak RH Oxnard GR Targeted therapy as an alternative to whole-brain radiotherapy in EGFR-mutant or ALK-positive: non–small-cell lung cancer with brain metastases. JAMA Oncol. (2017) 3:1274–5. 10.1001/jamaoncol.2017.1047 28520828

[30] Zhou L Deng L Lu Y Epidermal growth factor receptor mutations in non-small-cell lung cancer with brain metastasis: can up-front radiation therapy be deferred or withheld? J Clin Oncol. (2017) 35:1033–35. 10.1200/JCO.2016.71.5706 28113018

[31] Khandekar MJ Piotrowska Z Willers H Sequist LV. Role of epidermal growth factor receptor (EGFR) inhibitors and radiation in the management of brain metastases from EGFR mutant lung cancers. Oncologist (2018) 23:1054–62. 10.1634/theoncologist.2017-0557 29703765 PMC6192617

[32] Zhuang H Wang J Zhao L Yuan Z Wang P. The theoretical foundation and research progress for WBRT combined with erlotinib for the treatment of multiple brain metastases in patients with lung adenocarcinoma. Int J Cancer (2013) 133:2277–83. 10.1002/ijc.28290 23720067

[33] Fonkalsrud EW Sanchez M Zerubavel R Mahoney A. Serial changes in arterial structure following radiation therapy. Surg Gynecol Obstet. (1977) 145:395–400. 888060

[34] Reinhold HS Buisman GH. Radiosensitivity of capillary endothelium. Br J Radiol. (1973) 46:54–7. 4683331 10.1259/0007-1285-46-541-54

[35] Jiang T Min W Li Y Yue Z Wu C Zhou C. Radiotherapy plus EGFR TKIs in non-small cell lung cancer patients with brain metastases: an update meta-analysis. Cancer Med. (2016) 5:1055–65. 10.1002/cam4.673 26990668 PMC4924363

[36] Di Lorenzo R Ahluwalia MS. Targeted therapy of brain metastases: latest evidence and clinical implications. Ther Adv Med Oncol. (2017) 9:781–96. 10.1177/1758834017736252 29449898 PMC5808839

