# Supplementary material for: Upfront Cranial Radiotherapy vs. EGFR Tyrosine Kinase Inhibitors Alone for the Treatment of Brain Metastases From Non-small-cell Lung Cancer: A Meta-Analysis of 1465 Patients
Source: Front Oncol. 2018 Dec 12;8:603. doi: 10.3389/fonc.2018.00603 (PMC6299879; doi:10.3389/fonc.2018.00603)
Supplement: Table S1 — Quality assessment of 12 retrospective studies using the Newcastle-Ottawa scale. [file Table_1.docx]

**Table S1.** Quality assessment of 12 retrospective studies using the Newcastle-Ottawa scale

| **Study** | **Selection** | | | | **Comparability** | **Outcome** | | | **Quality Score** |
| --- | --- | --- | --- | --- | --- | --- | --- | --- | --- |
|  | Representativeness of the Exposed Cohort (1) | Selection of the Non-Exposed Cohort (1) | Ascertainment of Exposure (1) | Demonstration That Outcome of Interest Was Not Present at Start of Study (1) | Comparability of Cohorts on the Basis of the Design or Analysis (2) | Assessment of Outcome (1) | Was Follow-Up Long Enough for Outcomes to Occur (1) | Adequacy of Follow Up of Cohorts (1) |  |
| Zeng 2012 | 1 | 1 | 1 | 0 | 0 | 1 | 1 | 1 | 6 |
| Gerber 2014 | 1 | 1 | 1 | 0 | 1 | 1 | 1 | 1 | 7 |
| Byeon 2016 | 1 | 1 | 1 | 0 | 1 | 1 | 1 | 1 | 7 |
| Chen 2016 | 1 | 1 | 1 | 0 | 1 | 1 | 1 | 1 | 7 |
| Jiang 2016 | 1 | 1 | 1 | 0 | 0 | 1 | 0 | 1 | 5 |
| Magnuson 2016 | 1 | 1 | 1 | 0 | 1 | 1 | 1 | 1 | 7 |
| Fan 2017 | 1 | 1 | 1 | 0 | 1 | 1 | 1 | 1 | 7 |
| Liu 2017 | 1 | 1 | 1 | 0 | 0 | 1 | 1 | 1 | 6 |
| Magnuson 2017 | 1 | 1 | 1 | 0 | 1 | 1 | 1 | 1 | 7 |
| Zhu 2017 | 1 | 1 | 1 | 0 | 1 | 1 | 1 | 1 | 7 |
| Li 2018 | 1 | 1 | 1 | 0 | 1 | 1 | 1 | 1 | 7 |
| Sung 2018 | 1 | 1 | 1 | 0 | 1 | 1 | 1 | 1 | 7 |
