# Supplementary material for: Upfront Cranial Radiotherapy vs. EGFR Tyrosine Kinase Inhibitors Alone for the Treatment of Brain Metastases From Non-small-cell Lung Cancer: A Meta-Analysis of 1465 Patients
Source: Front Oncol. 2018 Dec 12;8:603. doi: 10.3389/fonc.2018.00603 (PMC6299879; doi:10.3389/fonc.2018.00603)
Supplement: Table S2 — Quality assessment of one randomized controlled trial. [file Table_2.docx]

**Table S2.** Quality assessment of one randomized controlled trial

| **Study** | **Random sequence generation** | **Allocation concealment** | **Blinding** | **Incomplete outcome data** | **Selective reporting** | **Other bias** | **Study quality** |
| --- | --- | --- | --- | --- | --- | --- | --- |
| Yang 2017 | Low risk | Unclear risk | High risk | Low risk | Low risk | Low risk | C |
