# Supplementary material for: Upfront Cranial Radiotherapy vs. EGFR Tyrosine Kinase Inhibitors Alone for the Treatment of Brain Metastases From Non-small-cell Lung Cancer: A Meta-Analysis of 1465 Patients
Source: Front Oncol. 2018 Dec 12;8:603. doi: 10.3389/fonc.2018.00603 (PMC6299879; doi:10.3389/fonc.2018.00603)
Supplement: Table S3 — Comparing the tumor burden between WBRT group and SRS group. [file Table_3.DOCX]

**Table S3.** Comparing the tumor burden between WBRT group and SRS group

| **Study** | **Intracranial tumor burden:**  **multiple BMs*** | | **Intracranial tumor burden:**  **largest BM＞1cm** | |
| --- | --- | --- | --- | --- |
|  | **WBRT** | **SRS** | **WBRT** | **SRS** |
| Gerber,2014 | 88% | 20% | 84% | 73% |
| Magnuson, 2017 | 74% | 18% | 65% | 56% |

Abbreviations: BMs, brain metastases; WBRT, whole brain radiotherapy; SRS, stereotactic radiosurgery;

*Multiple BMs indicated more than 3 metastases presented in brain for Gerber’s study and more than 4 for Magnuson’s study in 2017.
