# Supplementary material for: Upfront Cranial Radiotherapy vs. EGFR Tyrosine Kinase Inhibitors Alone for the Treatment of Brain Metastases From Non-small-cell Lung Cancer: A Meta-Analysis of 1465 Patients
Source: Front Oncol. 2018 Dec 12;8:603. doi: 10.3389/fonc.2018.00603 (PMC6299879; doi:10.3389/fonc.2018.00603)
Supplement: Table S4 — Detailed characteristics for each included study. [file Table_4.DOCX]

**Table S4.** Detailed characteristics for each included study

| **Study** | **Brain imaging** | **Symptomatic BMs** | | **Intracranial tumor burden:**  **multiple BMs*** | | **Intracranial tumor burden:**  **largest BM＞1cm** | | **Severity of disease：**  **GPA0-2** | | **Severity of disease：**  **Extracranial metastases** | | **Prior brain surgery** | |
| --- | --- | --- | --- | --- | --- | --- | --- | --- | --- | --- | --- | --- | --- |
|  |  | RT group | TKI group | RT group | TKI group | RT group | TKI group | RT group | TKI group | RT group | TKI group | RT group | TKI group |
| Gerber,2014 | MRI | 31% | 3% | 88% | 48% | 84% | 27% | 59% | 64% |  |  | 2/32 | 1/63 |
| Chen, 2016 | CT/MRI | 69.8% | 34.4% | 79.2% | 69.6% |  |  |  |  |  |  | 0 | 0 |
| Jiang, 2016 | CT/MRI |  |  |  |  |  |  |  |  |  |  |  |  |
| Magnuson, 2017 | MRI | 51% | 12% | 74% | 36% | 65% | 34% | 75% | 60% | 22% | 22% | 0 | 0 |
| Yang, 2017 | CT/MRI | 18% | 15% | 100% | 100% |  |  |  |  | 27% | 22% | 0 | 0 |
| Li, 2018 | CT/MRI | 58.8% | 9.1% | 35.3% | 54.5% |  |  |  |  |  |  | 0 | 0 |
| Zeng, 2012 | MRI |  |  |  |  | 100% | 100% |  |  |  |  | 0 | 0 |
| Byeon, 2016 | MRI | 48% | 20% | 64% | 76% |  |  |  |  | 68% | 93% | 0 | 0 |
| Magnuson, 2016 | MRI | 54% | 6% | 58% | 35% | 58% | 35% | 61% | 47% | 54% | 53% | 0 | 0 |
| Fan, 2017 | CT/MRI | 37% | 12% | 59% | 34% |  |  | 75% | 73% | 61% | 90% | 9/56 | 0/41 |
| Liu, 2017 | MRI |  |  |  |  |  |  | 100% | 100% |  |  | 0 | 0 |
| Zhu, 2017 | MRI |  |  | 73.1% | 65.2% |  |  |  |  | 65.7% | 75.2% |  |  |
| Sung, 2018 | MRI | 20% | 2.4% | 37.5% | 46.3% | 57.5% | 26.8% |  |  |  |  | 0 | 0 |

Abbreviations: BMs, brain metastases; GPA, graded prognostic assessment; RT, radiotherapy; TKI, tyrosine kinase inhibitors;

*Multiple BMs indicated more than 3 metastases presented in brain for most of the studies. But the number of BMs was set as 4 for Magnuson’s study in 2017, and 5 for Byeon’s study in 2016.
