# Supplementary figures and images for: Upfront Cranial Radiotherapy vs. EGFR Tyrosine Kinase Inhibitors Alone for the Treatment of Brain Metastases From Non-small-cell Lung Cancer: A Meta-Analysis of 1465 Patients
Source: Front Oncol. 2018 Dec 12;8:603. doi: 10.3389/fonc.2018.00603 (PMC6299879; doi:10.3389/fonc.2018.00603)

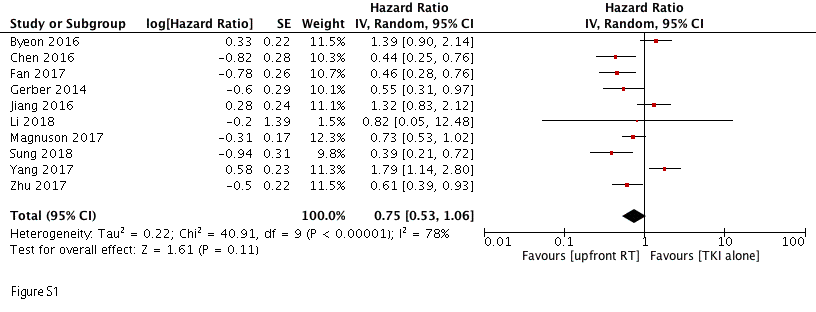

Supplement: Figure S1 — Forest plot and meta-analysis of intacranial progression-free survival (PFS) comparing upfront RT with TKI alone. RT, radiotherapy; TKI, tyrosine kinase inhibitor. [file Image_1.TIF]
